# Poly[diaqua­bis­[μ-1-hy­droxy-2-(imidazol-3-ium-1-yl)ethane-1,1-diyldiphospho­nato]tricopper(II)]

**DOI:** 10.1107/S1600536810046398

**Published:** 2010-11-17

**Authors:** Yaping Li, Dajun Sun, Hu Zang, Liying Han, Guanfang Su

**Affiliations:** a Department of Ophthalmology, The Second Hospital of Jilin University, Changchun 130041, People’s Republic of China; b Department of Vascular Surgery, The China–Japan Union Hospital of Jilin University, Changchun 130041, People’s Republic of China; c Department of Orthopedics, The China–Japan Union Hospital of Jilin University, Changchun 130041, People’s Republic of China; d Department of Gynecology, The Second Hospital of Jilin University, Changchun 130041, People’s Republic of China

## Abstract

In the title coordination polymer, [Cu_3_(C_5_H_7_N_2_O_7_P_2_)_2_(H_2_O)_2_]_*n*_, one Cu^II^ atom is five-coordinated by five O atoms from three 1-hy­droxy-2-(imidazol-3-ium-1-yl)ethane-1,1-diyldiphospho­nate (*L*) ligands in a distorted square-pyramidal geometry. The other Cu^II^ atom, lying on an inversion center, is six-coordinated in a distorted octa­hedral geometry by four O atoms from two *L* ligands and two O atoms from two water mol­ecules. The five-coordinated Cu^II^ atoms are linked by phospho­nate O atoms of the *L* ligands, forming a polymeric chain. These chains are further linked by the six-coordinated Cu atoms into a layer parallel to (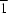
01). N—H⋯O and O—H⋯O hydrogen bonds connect the layers into a three-dimensional supra­molecular structure.

## Related literature

For general background to the applications of metal phospho­nates, see: Katz *et al.* (1994 ▶).
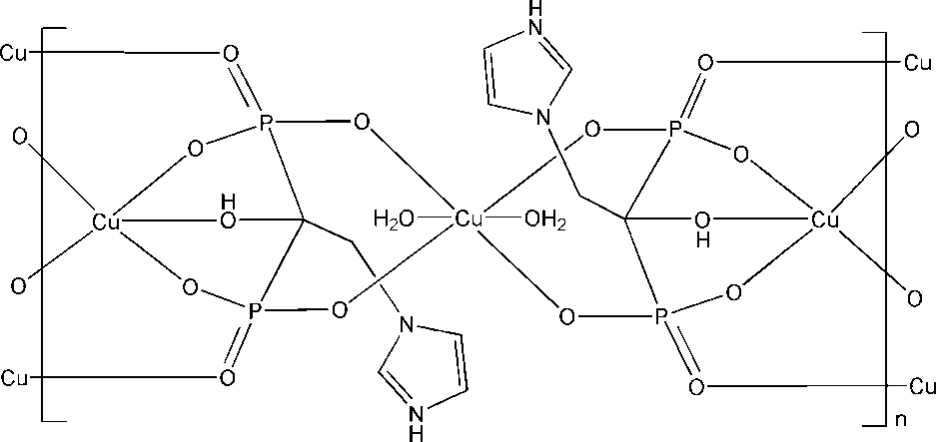


## Experimental

### 

#### Crystal data

[Cu_3_(C_5_H_7_N_2_O_7_P_2_)_2_(H_2_O)_2_]
*M* *_r_* = 764.81
Triclinic, 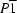

*a* = 7.4167 (9) Å
*b* = 8.1502 (10) Å
*c* = 9.5228 (12) Å
α = 104.747 (2)°
β = 107.658 (2)°
γ = 101.484 (2)°
*V* = 506.03 (11) Å^3^
*Z* = 1
Mo *K*α radiation
μ = 3.54 mm^−1^
*T* = 293 K
0.30 × 0.28 × 0.21 mm

#### Data collection

Bruker APEX CCD diffractometer
Absorption correction: multi-scan (*SADABS*; Sheldrick, 1996 ▶) *T*_min_ = 0.58, *T*_max_ = 0.75
2771 measured reflections
1973 independent reflections
1729 reflections with *I* > 2σ(*I*)
*R*_int_ = 0.012

#### Refinement

*R*[*F* ^2^ > 2σ(*F* ^2^)] = 0.027
*wR*(*F* ^2^) = 0.073
*S* = 1.05
1973 reflections
175 parameters
2 restraints
H atoms treated by a mixture of independent and constrained refinement
Δρ_max_ = 0.55 e Å^−3^
Δρ_min_ = −0.68 e Å^−3^

### 

Data collection: *SMART* (Bruker, 2007 ▶); cell refinement: *SAINT* (Bruker, 2007 ▶); data reduction: *SAINT*; program(s) used to solve structure: *SHELXS97* (Sheldrick, 2008 ▶); program(s) used to refine structure: *SHELXL97* (Sheldrick, 2008 ▶); molecular graphics: *SHELXTL* (Sheldrick, 2008 ▶) and *DIAMOND* (Brandenburg, 1999 ▶); software used to prepare material for publication: *SHELXTL*.

## Supplementary Material

Crystal structure: contains datablocks global, I. DOI: 10.1107/S1600536810046398/hy2377sup1.cif

Structure factors: contains datablocks I. DOI: 10.1107/S1600536810046398/hy2377Isup2.hkl

Additional supplementary materials:  crystallographic information; 3D view; checkCIF report

## Figures and Tables

**Table 1:** Hydrogen-bond geometry (Å, °)

*D*—H⋯*A*	*D*—H	H⋯*A*	*D*⋯*A*	*D*—H⋯*A*
N2—H2*A*⋯O6^i^	0.86	1.94	2.771 (4)	163
O7—H7⋯O4	0.82	2.16	2.724 (3)	126
O1*W*—H1*A*⋯O3^ii^	0.88 (5)	2.09 (3)	2.921 (4)	157 (5)
O1*W*—H1*B*⋯O2^iii^	0.87 (2)	2.13 (4)	2.851 (4)	140 (4)

Symmetry codes: (i) 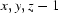; (ii) 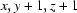; (iii) 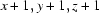.

**Fig. 1.**
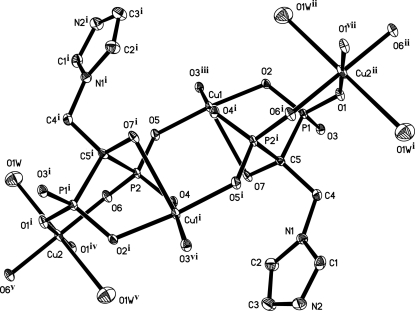
Structure of the title compound. Displacement ellipsoids are drawn at the 50% probability level. H atoms have been omitted for clarity. [Symmetry codes: (i) -x, -y - 1, -z; (ii) x - 1, y - 1, z - 1; (iii) -x, -y - 2, -z; (iv) x + 1, y + 1, z + 1; (v) -x + 1, -y, -z + 1; (vi) x, y + 1, z; (vii) -x - 1, -y - 2, -z - 1.]

**Fig. 2.**
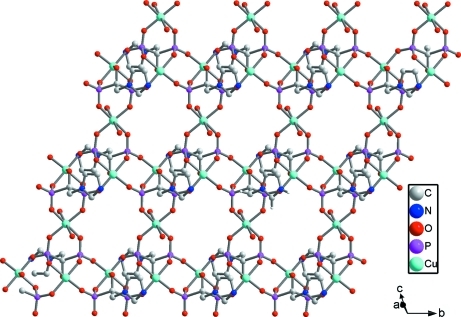
Two-dimensional layer structure in the title compound.

## supplementary crystallographic information

### Comment

During the last two decades great research efforts have been devoted to the synthesis and design of metal phosphonates due to their potential applications in electrooptics, ion exchange, catalysis, and stent in intestinal or biliary (Katz *et al.*, 1994). Herein, we present a new copper(II)–phosphonate complex.

The structure analysis reveals that the title compound has a two-dimensional polymeric structure. As shown in Fig. 1, there exist two kinds of crystallographically unique Cu^II^ ions. Atom Cu1 is five-coordinated by four phosphonate O atoms and one hydroxy O atom from three 2-(imidazol-3-ium-1-yl)-1-hydroxy-1,1-ethylidenediphosphonate (*L*) ligands. Atom Cu2 is six-coordinated by four O atoms from two *L* ligands and two O atoms from two water molecules. The Cu1 atoms are linked by the phosphonate O atoms, resulting in a one-dimensional polymeric chain. These chains are further linked by the Cu2 atoms into a layer (Fig. 2). N—H···O and O—H···O hydrogen bonds involving the coordinated water molecules and *L* ligands (Table 1) lead to the formation of a three-dimensional supramolecular network.

### Experimental

The synthesis was performed under hydrothermal conditions. A mixture of CuCl_2_.2H_2_O (0.034 g, 0.2 mmol), *L* ligand (0.070 g, 0.2 mmol) and H_2_O (15 ml) in a 25 ml stainless steel reactor with a Teflon liner was heated from 293 to 423 K in 2 h and maintained at 423 K for 72 h. After the mixture was cooled to 298 K, green crystals of the title compound were obtained (yield: 56%).

### Refinement

H atoms bound to C, N and hydroxy O were positioned geometrically and refined using a riding model, with C—H = 0.93 and 0.97, N—H = 0.86 and O—H = 0.82 Å and with *U*_iso_(H) = 1.2(1.5 for hydroxy)*U*_eq_(C,*N*,*O*). H atoms of water molecules were located in a difference Fourier map and refined with *U*_iso_(H) = 1.5*U*_eq_(O).

### Crystal data

**Table:**

[Cu_3_(C_5_H_7_N_2_O_7_P_2_)_2_(H_2_O)_2_]	*Z* = 1
*M**_r_* = 764.81	*F*(000) = 381
Triclinic, *P*1	*D*_x_ = 2.510 Mg m^−^^3^
Hall symbol: -P 1	Mo *K*α radiation, λ = 0.71073 Å
*a* = 7.4167 (9) Å	Cell parameters from 1973 reflections
*b* = 8.1502 (10) Å	θ = 1.9–28.3°
*c* = 9.5228 (12) Å	µ = 3.54 mm^−^^1^
α = 104.747 (2)°	*T* = 293 K
β = 107.658 (2)°	Block, blue
γ = 101.484 (2)°	0.30 × 0.28 × 0.21 mm
*V* = 506.03 (11) Å^3^	

### Data collection

**Table:**

Bruker APEX CCD diffractometer	1973 independent reflections
Radiation source: fine-focus sealed tube	1729 reflections with *I* > 2σ(*I*)
graphite	*R*_int_ = 0.012
φ and ω scans	θ_max_ = 26.1°, θ_min_ = 2.4°
Absorption correction: multi-scan (*SADABS*; Sheldrick, 1996)	*h* = −9→8
*T*_min_ = 0.58, *T*_max_ = 0.75	*k* = −10→6
2771 measured reflections	*l* = −11→11

### Refinement

**Table:**

Refinement on *F*^2^	Primary atom site location: structure-invariant direct methods
Least-squares matrix: full	Secondary atom site location: difference Fourier map
*R*[*F*^2^ > 2σ(*F*^2^)] = 0.027	Hydrogen site location: inferred from neighbouring sites
*wR*(*F*^2^) = 0.073	H atoms treated by a mixture of independent and constrained refinement
*S* = 1.05	*w* = 1/[σ^2^(*F*_o_^2^) + (0.039*P*)^2^ + 0.8352*P*] where *P* = (*F*_o_^2^ + 2*F*_c_^2^)/3
1973 reflections	(Δ/σ)_max_ = 0.001
175 parameters	Δρ_max_ = 0.55 e Å^−^^3^
2 restraints	Δρ_min_ = −0.68 e Å^−^^3^

### Fractional atomic coordinates and isotropic or equivalent isotropic displacement parameters (Å^2^)

**Table:**

	*x*	*y*	*z*	*U*_iso_*/*U*_eq_	
C1	0.2068 (5)	−0.5648 (4)	−0.4517 (4)	0.0140 (7)	
H1	0.1047	−0.6119	−0.5497	0.017*	
C2	0.3998 (5)	−0.5281 (5)	−0.2131 (4)	0.0173 (7)	
H2	0.4524	−0.5475	−0.1190	0.021*	
C3	0.4782 (5)	−0.3932 (5)	−0.2542 (4)	0.0186 (7)	
H3	0.5935	−0.2995	−0.1928	0.022*	
C4	0.0950 (5)	−0.7988 (4)	−0.3483 (4)	0.0122 (7)	
H4A	0.1673	−0.8856	−0.3460	0.015*	
H4B	−0.0147	−0.8435	−0.4487	0.015*	
C5	0.0110 (5)	−0.7833 (4)	−0.2191 (4)	0.0090 (6)	
N1	0.2266 (4)	−0.6324 (4)	−0.3368 (3)	0.0110 (6)	
N2	0.3567 (4)	−0.4195 (4)	−0.4031 (3)	0.0159 (6)	
H2A	0.3750	−0.3518	−0.4568	0.019*	
O1	−0.2890 (3)	−1.0888 (3)	−0.3981 (2)	0.0104 (5)	
O2	−0.2018 (3)	−0.9799 (3)	−0.1041 (2)	0.0098 (5)	
O3	0.0217 (3)	−1.1193 (3)	−0.2195 (2)	0.0099 (5)	
O4	0.2060 (3)	−0.3636 (3)	0.0771 (2)	0.0100 (5)	
O5	0.0648 (3)	−0.5288 (3)	0.2312 (2)	0.0105 (5)	
O6	0.3438 (3)	−0.2484 (3)	0.3787 (3)	0.0115 (5)	
O7	0.1644 (3)	−0.7118 (3)	−0.0644 (2)	0.0105 (5)	
H7	0.2315	−0.6121	−0.0511	0.016*	
P1	−0.12407 (12)	−1.01022 (10)	−0.23716 (9)	0.00780 (18)	
P2	0.16460 (12)	−0.34839 (10)	0.22756 (9)	0.00806 (18)	
Cu1	−0.07744 (6)	−0.75596 (5)	0.06756 (4)	0.00871 (12)	
Cu2	0.5000	0.0000	0.5000	0.01057 (15)	
O1W	0.3909 (4)	−0.0271 (4)	0.7275 (3)	0.0282 (6)	
H1A	0.302 (6)	−0.054 (7)	0.768 (5)	0.042*	
H1B	0.491 (5)	−0.001 (6)	0.815 (4)	0.042*	

### Atomic displacement parameters (Å^2^)

**Table:**

	*U*^11^	*U*^22^	*U*^33^	*U*^12^	*U*^13^	*U*^23^
C1	0.0184 (18)	0.0150 (17)	0.0093 (15)	0.0056 (14)	0.0050 (13)	0.0048 (13)
C2	0.0147 (17)	0.0198 (18)	0.0129 (16)	0.0011 (14)	0.0017 (14)	0.0058 (14)
C3	0.0171 (18)	0.0175 (18)	0.0163 (17)	−0.0001 (14)	0.0032 (14)	0.0056 (14)
C4	0.0152 (17)	0.0084 (15)	0.0117 (16)	0.0021 (13)	0.0061 (13)	0.0011 (13)
C5	0.0099 (15)	0.0080 (15)	0.0064 (14)	−0.0002 (12)	0.0010 (12)	0.0026 (12)
N1	0.0119 (13)	0.0090 (13)	0.0128 (13)	0.0032 (11)	0.0050 (11)	0.0040 (11)
N2	0.0209 (16)	0.0133 (15)	0.0156 (14)	0.0029 (12)	0.0077 (12)	0.0089 (12)
O1	0.0133 (12)	0.0068 (11)	0.0075 (11)	0.0025 (9)	0.0004 (9)	0.0009 (9)
O2	0.0126 (11)	0.0060 (11)	0.0093 (10)	0.0004 (9)	0.0045 (9)	0.0019 (9)
O3	0.0137 (11)	0.0072 (11)	0.0089 (11)	0.0032 (9)	0.0036 (9)	0.0032 (9)
O4	0.0134 (11)	0.0080 (11)	0.0091 (11)	0.0042 (9)	0.0044 (9)	0.0029 (9)
O5	0.0149 (12)	0.0075 (11)	0.0072 (11)	0.0021 (9)	0.0028 (9)	0.0020 (9)
O6	0.0127 (11)	0.0082 (11)	0.0086 (11)	0.0016 (9)	−0.0003 (9)	0.0013 (9)
O7	0.0107 (11)	0.0075 (11)	0.0077 (11)	−0.0004 (9)	−0.0008 (9)	0.0008 (9)
P1	0.0101 (4)	0.0055 (4)	0.0064 (4)	0.0017 (3)	0.0022 (3)	0.0014 (3)
P2	0.0104 (4)	0.0049 (4)	0.0062 (4)	0.0012 (3)	0.0014 (3)	0.0006 (3)
Cu1	0.0123 (2)	0.0056 (2)	0.0063 (2)	0.00188 (15)	0.00214 (15)	0.00108 (15)
Cu2	0.0109 (3)	0.0053 (3)	0.0098 (3)	0.0012 (2)	−0.0012 (2)	0.0005 (2)
O1W	0.0233 (15)	0.0366 (17)	0.0267 (15)	0.0095 (13)	0.0093 (12)	0.0132 (13)

### Geometric parameters (Å, °)

**Table:**

C1—N2	1.318 (4)	O2—Cu1	1.936 (2)
C1—N1	1.329 (4)	O3—P1	1.529 (2)
C1—H1	0.9300	O3—Cu1^iii^	1.962 (2)
C2—C3	1.343 (5)	O4—P2	1.534 (2)
C2—N1	1.377 (4)	O4—Cu1^i^	2.003 (2)
C2—H2	0.9300	O5—P2	1.523 (2)
C3—N2	1.364 (4)	O5—Cu1	1.930 (2)
C3—H3	0.9300	O6—P2	1.521 (2)
C4—N1	1.462 (4)	O6—Cu2	1.959 (2)
C4—C5	1.528 (4)	O7—H7	0.8200
C4—H4A	0.9700	P2—C5^i^	1.842 (3)
C4—H4B	0.9700	Cu1—O3^iii^	1.962 (2)
C5—O7	1.444 (4)	Cu1—O4^i^	2.003 (2)
C5—P2^i^	1.842 (3)	Cu2—O1^i^	1.950 (2)
C5—P1	1.857 (3)	Cu2—O1^iv^	1.950 (2)
N2—H2A	0.8600	Cu2—O6^v^	1.959 (2)
O1—P1	1.519 (2)	O1W—H1A	0.88 (5)
O1—Cu2^ii^	1.950 (2)	O1W—H1B	0.87 (2)
O2—P1	1.530 (2)		
			
N2—C1—N1	108.3 (3)	P2—O4—Cu1^i^	119.28 (13)
N2—C1—H1	125.8	P2—O5—Cu1	131.88 (14)
N1—C1—H1	125.8	P2—O6—Cu2	136.89 (14)
C3—C2—N1	107.0 (3)	C5—O7—H7	109.5
C3—C2—H2	126.5	O1—P1—O3	111.33 (12)
N1—C2—H2	126.5	O1—P1—O2	112.88 (13)
C2—C3—N2	107.0 (3)	O3—P1—O2	112.51 (12)
C2—C3—H3	126.5	O1—P1—C5	106.82 (13)
N2—C3—H3	126.5	O3—P1—C5	108.50 (14)
N1—C4—C5	114.6 (3)	O2—P1—C5	104.30 (13)
N1—C4—H4A	108.6	O6—P2—O5	109.75 (13)
C5—C4—H4A	108.6	O6—P2—O4	114.99 (13)
N1—C4—H4B	108.6	O5—P2—O4	112.22 (12)
C5—C4—H4B	108.6	O6—P2—C5^i^	107.03 (13)
H4A—C4—H4B	107.6	O5—P2—C5^i^	108.36 (14)
O7—C5—C4	112.5 (3)	O4—P2—C5^i^	104.02 (13)
O7—C5—P2^i^	108.6 (2)	O5—Cu1—O2	174.89 (9)
C4—C5—P2^i^	114.1 (2)	O5—Cu1—O3^iii^	91.03 (9)
O7—C5—P1	105.3 (2)	O2—Cu1—O3^iii^	91.03 (9)
C4—C5—P1	108.5 (2)	O5—Cu1—O4^i^	90.70 (9)
P2^i^—C5—P1	107.27 (16)	O2—Cu1—O4^i^	88.63 (9)
C1—N1—C2	108.3 (3)	O3^iii^—Cu1—O4^i^	163.80 (9)
C1—N1—C4	124.8 (3)	O1^i^—Cu2—O1^iv^	180.00 (13)
C2—N1—C4	126.7 (3)	O1^i^—Cu2—O6	92.58 (9)
C1—N2—C3	109.3 (3)	O1^iv^—Cu2—O6	87.42 (9)
C1—N2—H2A	125.3	O1^i^—Cu2—O6^v^	87.42 (9)
C3—N2—H2A	125.3	O1^iv^—Cu2—O6^v^	92.58 (9)
P1—O1—Cu2^ii^	131.22 (13)	O6—Cu2—O6^v^	180.00 (19)
P1—O2—Cu1	118.08 (13)	H1A—O1W—H1B	93 (4)
P1—O3—Cu1^iii^	125.96 (13)		
			
N1—C2—C3—N2	−2.0 (4)	P2^i^—C5—P1—O1	63.64 (18)
N1—C4—C5—O7	−56.4 (4)	O7—C5—P1—O3	−60.7 (2)
N1—C4—C5—P2^i^	67.9 (3)	C4—C5—P1—O3	60.0 (2)
N1—C4—C5—P1	−172.6 (2)	P2^i^—C5—P1—O3	−176.24 (13)
N2—C1—N1—C2	−2.1 (4)	O7—C5—P1—O2	59.4 (2)
N2—C1—N1—C4	−176.7 (3)	C4—C5—P1—O2	−179.9 (2)
C3—C2—N1—C1	2.5 (4)	P2^i^—C5—P1—O2	−56.12 (17)
C3—C2—N1—C4	177.0 (3)	Cu2—O6—P2—O5	−156.45 (19)
C5—C4—N1—C1	−127.4 (3)	Cu2—O6—P2—O4	75.9 (2)
C5—C4—N1—C2	58.9 (4)	Cu2—O6—P2—C5^i^	−39.1 (2)
N1—C1—N2—C3	0.9 (4)	Cu1—O5—P2—O6	−147.75 (17)
C2—C3—N2—C1	0.7 (4)	Cu1—O5—P2—O4	−18.6 (2)
Cu2^ii^—O1—P1—O3	−172.39 (16)	Cu1—O5—P2—C5^i^	95.7 (2)
Cu2^ii^—O1—P1—O2	60.0 (2)	Cu1^i^—O4—P2—O6	−115.31 (15)
Cu2^ii^—O1—P1—C5	−54.1 (2)	Cu1^i^—O4—P2—O5	118.31 (14)
Cu1^iii^—O3—P1—O1	−118.20 (16)	Cu1^i^—O4—P2—C5^i^	1.40 (18)
Cu1^iii^—O3—P1—O2	9.7 (2)	P2—O5—Cu1—O3^iii^	156.80 (19)
Cu1^iii^—O3—P1—C5	124.53 (16)	P2—O5—Cu1—O4^i^	−39.31 (19)
Cu1—O2—P1—O1	−134.92 (14)	P1—O2—Cu1—O3^iii^	−124.84 (14)
Cu1—O2—P1—O3	98.05 (15)	P1—O2—Cu1—O4^i^	71.36 (15)
Cu1—O2—P1—C5	−19.34 (18)	P2—O6—Cu2—O1^i^	19.3 (2)
O7—C5—P1—O1	179.20 (18)	P2—O6—Cu2—O1^iv^	−160.7 (2)
C4—C5—P1—O1	−60.1 (2)		

Symmetry codes: (i) −*x*, −*y*−1, −*z*; (ii) *x*−1, *y*−1, *z*−1; (iii) −*x*, −*y*−2, −*z*; (iv) *x*+1, *y*+1, *z*+1; (v) −*x*+1, −*y*, −*z*+1.

### Hydrogen-bond geometry (Å, °)

**Table:**

*D*—H···*A*	*D*—H	H···*A*	*D*···*A*	*D*—H···*A*
N2—H2A···O6^vi^	0.86	1.94	2.771 (4)	163
O7—H7···O4	0.82	2.16	2.724 (3)	126
O1W—H1A···O3^vii^	0.88 (5)	2.09 (3)	2.921 (4)	157 (5)
O1W—H1B···O2^iv^	0.87 (2)	2.13 (4)	2.851 (4)	140 (4)

Symmetry codes: (vi) *x*, *y*, *z*−1; (vii) *x*, *y*+1, *z*+1; (iv) *x*+1, *y*+1, *z*+1.
